# Supplementary material for: Preoperative Hilar and Mediastinal Lymph Node Staging in Patients with Suspected or Diagnosed Lung Cancer: Accuracy of 18F-FDG-PET/CT:A Retrospective Cohort Study of 138 Patients
Source: Diagnostics (Basel). 2023 Jan 22;13(3):403. doi: 10.3390/diagnostics13030403 (PMC9914665; doi:10.3390/diagnostics13030403)
Supplement: Supplementary file 1 [file diagnostics-13-00403-s001.zip › diagnostics-2043777-supplementary.pdf]

Table S1: Pattern of false negative HMLN involvement. (18 of 83 patients). LCNEC: Large cell neuroendocrine carcinoma, s/u: subtle uptake.

Case	Lobar distribution	Concurrent disease	Histological type	Tumor size (cm)	SUVmax of tumor	SUVmax of node	Pathological N(+) station
1	LUL	None	Adenocarcinoma	1.9	10.2	s/u	10, 11
2	LUL	Diabetes	Adenocarcinoma	3.0	6.5	s/u	10
3	RLL	None	Adenocarcinoma	1.8	3.6	s/u	2, 9, 11
4	LLL	None	Squamous cell carcinoma	4.8	8.87	s/u	10
5	LUL	COPD	Adenocarcinoma	4.0	4.5	s/u	12
6	LLL	Diabetes and COPD	Squamous cell carcinoma	4.8	11.8	s/u	7, 11
7	LLL	None	LCNEC	8.0	10.0	s/u	13
8	LUL	None	Adenocarcinoma	6.0	7.8	s/u	8
9	RUL	None	Adenocarcinoma	8.5	8.7	s/u	11
10	LLL	None	Squamous cell carcinoma	4.0	5.0	s/u	11
11	RML	None	Adenocarcinoma	2.8	22.8	s/u	11
12	RUL	None	Adenocarcinoma	2.4	24.3	s/u	11
13	RML	Diabetes	Squamous cell carcinoma	1.7	14.6	s/u	11
14	RUL	None	Adenocarcinoma	5.3	12.3	s/u	11
15	RUL	COPD	Adenocarcinoma	4.5	10.4	s/u	13
16	RLL	None	Squamous cell carcinoma	3.7	13.8	s/u	8
17	RLL	None	Adenocarcinoma	3.0	13	s/u	10
18	RUL	COPD	Adenocarcinoma	2.5	5.4	s/u	2, 4

Table S2: Pattern of false positive HMLN involvement. (29 of 55 patients). LCNEC: Large cell neuroendocrine carcinoma.

Case	Lobar distribution	Concurrent disease	Histological type	Tumor size (cm)	SUVmax of tumor	SUVmax of node	PET N Station
1	LUL	None	Adenocarcinoma	6.5	15.4	2.7	cN1
2	LUL	Diabetes	Squamous cell carcinoma	1.7	4.6	3.6	cN3
3	LUL	COPD	LCNEC	4.0	9.9	2.3	cN1
4	RLL	COPD	Squamous cell carcinoma	5.0	5.4	3.8	cN2
5	RUL	Diabetes	Adenocarcinoma	1.5	3.8	9.6	cN3
6	RUL	None	No malignancy	4.0	5.5	5.7	cN3
7	RML	None	No malignancy	1.5	3.1	3.1	cN2
8	RUL	COPD	Adenocarcinoma	3.2	7.5	2.8	cN3
9	RUL	None	Adenocarcinoma	6.0	15.3	2.5	cN1
10	LUL	COPD	Squamous cell carcinoma	0.5	3.7	2.7	cN1
11	RUL	COPD	Adenocarcinoma	1.3	9.9	8.9	cN3
12	LUL	None	No malignancy	1.3	7.8	9.3	cN3
13	RUL	None	No malignancy	1.2	8.0	3.4	cN2
14	LLL	COPD	Squamous cell carcinoma	1.3	9.1	7.5	cN3

15	RUL	COPD	Squamous cell carcinoma	6.0	13.2	4.4	cN1
16	RUL	COPD	Adenocarcinoma	5.3	2.2	3.5	cN3
17	LUL	None	Squamous cell carcinoma	7.5	12.7	3.5	cN2
18	RLL	COPD	Squamous cell carcinoma	1.3	5.4	3.2	cN2
19	LUL	None	Squamous cell carcinoma	2.8	17.6	3.4	cN1
20	LUL	COPD	Squamous cell carcinoma	8.0	11.9	4.2	cN3
21	RUL	None	Adenocarcinoma	2.1	3.7	3.1	cN3
22	RLL	None	Squamous cell carcinoma	6.6	13.4	3.0	cN1
23	RLL	COPD	Squamous cell carcinoma	2.9	22.8	2.7	cN1
24	LUL	COPD	Squamous cell carcinoma	3.4	12.9	3	cN2
25	LUL	COPD	Squamous cell carcinoma	0.8	3.6	3.8	cN3
26	RUL	None	No malignancy	6	16.5	4.5	cN2
27	RLL	COPD	LCNEC	4.6	32	11.2	cN2
28	RUL	None	Adenocarcinoma	4.5	13.3	3.2	cN2
29	LUL	None	Adenocarcinoma	3.7	34.7	3.6	cN1
